# Selling and Smooth-Talking: Effects of Interviewer Impression Management from a Signaling Perspective

**DOI:** 10.3389/fpsyg.2017.00740

**Published:** 2017-05-29

**Authors:** Annika Wilhelmy, Martin Kleinmann, Klaus G. Melchers, Martin Götz

**Affiliations:** ^1^Psychologisches Institut, Universität ZürichZürich, Switzerland; ^2^Institut für Psychologie und Pädagogik, Universität UlmUlm, Germany

**Keywords:** impression management, interview, applicant reactions, recruitment, signaling

## Abstract

Prior research suggests that interviewers play an important role in representing their organization and in making the interview a pleasant experience for applicants. This study examined whether impression management used by interviewers (organization-enhancement and applicant-enhancement) is perceived by applicants, and how it influences applicants' attitudes, intentions, and emotions. Adopting a signaling perspective, this article argues that applicants' positive attitudes and intentions toward the organization increase if interviewers not only enhance the organization, but if the signals they sent (i.e., organization-enhancement) are actually received by the applicant. Similarly, applicants' positive emotions should increase if interviewers not only enhance the applicant, but if the signals they send (i.e., applicant-enhancement) are actually received by the applicant. A field study that involved video coding interviewers' impression management behavior during 153 selection interviews and pre- and post-interview applicant surveys showed that the signals sent by interviewers during the interview were received by applicants. In addition, applicants rated the organization's prestige and their own positive affect after the interview more positively when they perceived higher levels of organization-enhancement during the interview. Furthermore, applicants reported more positive affect and interview self-efficacy after the interview when they perceived higher levels of interviewer applicant-enhancement. We also found an indirect effect of interviewers' organization-enhancement on organizational prestige through applicants' perceptions of organization-enhancement as well as indirect effects of interviewers' applicant-enhancement on applicants' positive affect and interview self-efficacy through applicants' perceptions of applicant-enhancement. Our findings contribute to an integrated understanding of the effects of interviewer impression management and point out both risks and chances in selling and smooth-talking toward applicants.

## Introduction

Over the last three decades, interviewers have gained a lot of research attention because of the important role they play in attracting applicants and thus in ensuring organizations' success. From the perspective of the interviewer, a successful interview is one in which the applicant is not only evaluated accurately, but also leaves the interview room with a favorable image of the organization and feeling good about him- or herself (Gilmore et al., 1999; Dipboye et al., 2012; Tsai and Huang, 2014). Research on interviewer impression management (IM)—defined as interviewers' attempts to influence the images applicants gain during social interactions (Schlenker, 1980)—suggests that the signals interviewers send to applicants have the potential to improve the effectiveness of recruitment activities (Stevens et al., 1990; Tsai and Huang, 2014; Wilhelmy et al., 2016). However, despite this initial proposition, we are still unclear about the mechanism by which the signals that are sent in terms of interviewer IM influence recruiting outcomes.

Scholars have repeatedly pointed out that it is crucial to study the mechanisms that explain how applicants respond to interviewers' IM signals. Doing so would provide a more comprehensive theoretical understanding of the effects of interviewer IM and offer recommendations to organizations on how to effectively use interviewer IM in recruitment (e.g., Gilmore et al., 1999; Celani and Singh, 2011; Tsai and Huang, 2014). Therefore, the aim of our study was to examine how interviewer IM influences recruiting outcomes by incorporating applicants' perceptions of interviewer IM: Are the signals that interviewers send actually received by applicants, and how do applicants react to the signals they receive? It is striking that past research has rarely addressed whether applicants are responsive at all to interviewers' use of IM, as prior studies have not differentiated between the signals that are sent (i.e., interviewer IM) and the signals that are received (i.e., applicants' perceptions of interviewer IM). In addition, previous research on the effects of interviewer IM has focused on laboratory settings even though applicants' perceptions and reactions can differ in real selection settings (Hausknecht et al., 2004; Chapman et al., 2005; Truxillo et al., 2009). Furthermore, past research has neglected the notion that applicants may already enter the interview with different attitudes, intentions, and emotions, and that these initial differences need to be considered in order to capture the influence of interviewer IM.

The current study extends existing research in several ways. First, we draw on signaling theory (Spence, 1973; Connelly et al., 2011; Bangerter et al., 2012) to provide the first study that examines how signals sent by interviewers in terms of IM influence recruiting outcomes. We develop and test a signaling timeline model of interviewer impression management and argue that for interviewer IM to be an effective means for recruitment, the signals sent (i.e., interviewer IM) need to be perceived by applicants (i.e., perceived interviewer IM), and applicants need to react to the signals they receive (i.e., recruiting outcomes). Second, we examine two interviewer goals that have repeatedly been emphasized in the literature: the goal of representing the organization to enhance applicants' positive attitudes and intentions toward the organization, and the goal of making applicants feel good in terms of applicants' positive emotions (Gilmore et al., 1999; Dipboye et al., 2012; Tsai and Huang, 2014; Wilhelmy et al., 2016). We link these goals to two interviewer IM behaviors that have been found to be particularly promising for recruitment purposes in past research: organization-enhancement, which seems to be particularly important to increase applicants' attitudes and intentions toward the organization, and applicant-enhancement, which seems particularly important to induce favorable emotional reactions in applicants (Stevens et al., 1990; Wilhelmy et al., 2016). Third, we extend prior research on interviewer IM by differentiating interviewer IM as signals sent (IM behavior that is applied by interviewers) vs. signals received (IM behavior that is perceived by applicants) by using different data sources such as video ratings of actual interviewer behavior and survey ratings on applicants' perceptions after the interview. Fourth, we extend prior research by examining interviewer IM in a high-stakes field setting instead of a laboratory setting. This is important because applicants' perceptions and reactions have been found to be different in real high-stakes settings compared to laboratory settings (Hausknecht et al., 2004; Chapman et al., 2005; Truxillo et al., 2009). Fifth, the current study considers applicants' initial attitudes, intentions, and emotions before the interview in addition to their attitudes, intentions, and emotions after the interview to extract the effects of interviewer IM. Finally, from a practical perspective, an integrated understanding of the effects of interviewer IM and the mechanisms explaining their effects is important for designing interviewer training programs. Interviewers could, for example, be trained on how to conduct interviews in a way that makes it a worthwhile experience for applicants and makes applicants feel attracted to the organization.

## Theory and hypotheses

**Figure 1:**
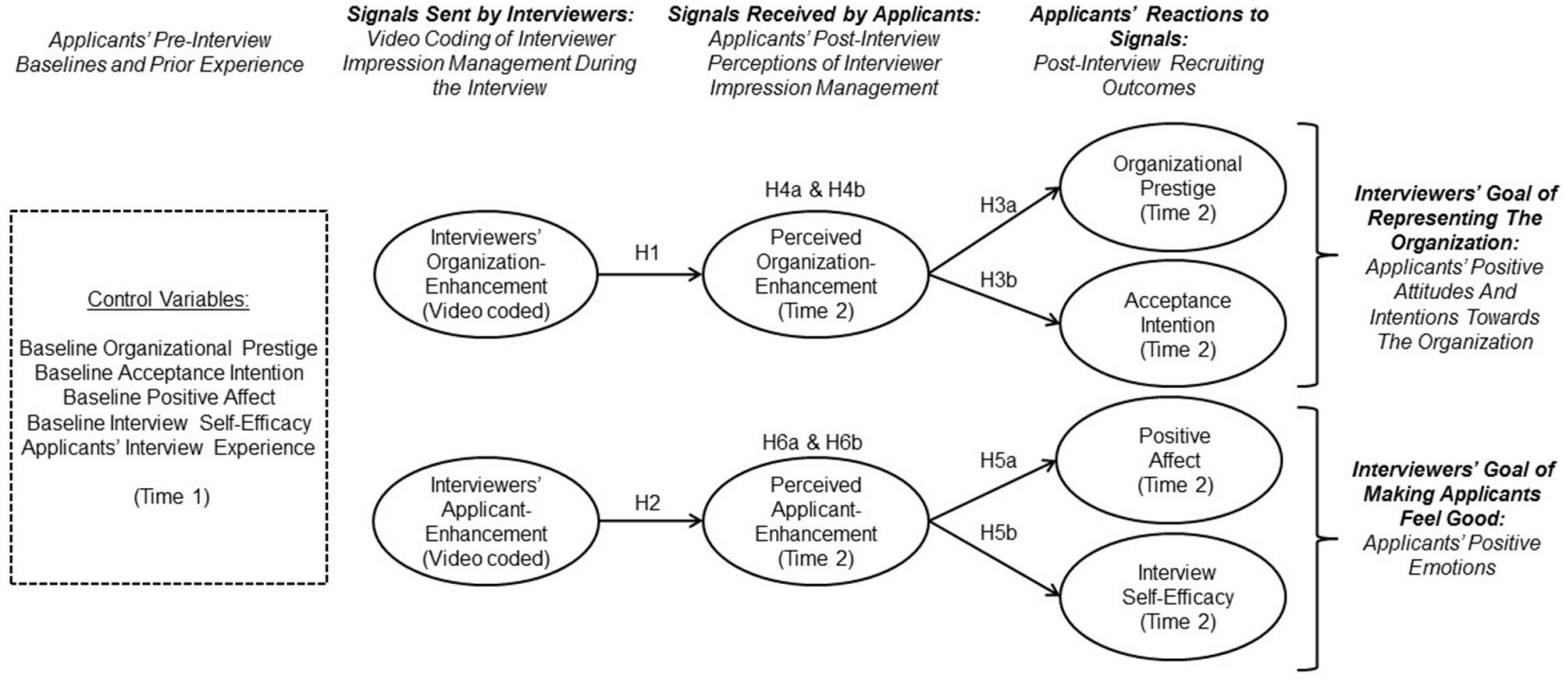
**Proposed signaling timeline model of impression management (IM): Interviewer IM (signals sent) are related to applicants' perceptions of interviewer IM (signals received), and indirectly, with recruiting outcomes (reactions to the signals received)**. Control variables are shown in the dashed box.

In the following sections, we delineate a signaling timeline model of the effects of interviewer IM. As shown in Figure 1, we propose that for interviewer IM to have an effect on recruiting outcomes, applicants need to first perceive interviewers' IM behavior, and then react to it. We also propose that organization-enhancement is particularly important for interviewers' goal of representing the organization in terms of increasing applicants' positive attitudes and intentions toward the organization. On the other hand, applicant-enhancement is particularly important for interviewers' goal of making applicants feel good in terms of increasing applicants' positive emotions. Below, we first present signaling theory as a theoretical rationale for these effects. We then review research on interviewer IM as signals sent and as signals received as well as research on recruitment effects of interviewer IM.

### Signaling theory

Signaling theory (Spence, 1973; Connelly et al., 2011; Bangerter et al., 2012) is widely used to explain how applicants' attraction to an organization can be influenced by information or signals during the recruitment and selection process. This theory suggests that the signals to which applicants are exposed have the potential to influence how applicants feel about themselves and the organization (Celani and Singh, 2011). When applicants receive information about the job and the work environment, it helps them decide whether or not they are excited about the job and whether they want to work for the organization (Farago et al., 2013). An important source from which applicants receive signals are employment interviews, which are characterized by a dynamic exchange between applicants and interviewers (Celani and Singh, 2011; Levashina et al., 2014).

Many theoretical frameworks suggest that the signals interviewers send to applicants influence applicants' attitudes, intentions, emotions, and behavioral reactions, but relatively little theoretical work has focused on applicants' perceptions of these signals. For example, as Celani and Singh (2011) point out, “an improved understanding of how applicants are influenced by recruitment signals will help to address outstanding research needs” (p. 231). Particularly, an issue that remains unresolved regarding signaling theory is how the signals that interviewers use to induce favorable reactions in applicants ultimately lead to these reactions.

The signaling timeline model by Connelly et al. (2011) specifies the process of how signals ultimately influence individual reactions, but has not been tested sufficiently in the field. According to the model, the most basic form of a signaling system consists of a sender (e.g., the interviewer), a signal (e.g., IM behavior), and a receiver (e.g., the applicant). In addition, the model suggests that there is a chronologic process through which signals have an effect: Signals are sent to the receiver, the receiver observes the signals, and eventually, the receiver reacts to the signals, for example, with an attitude, a behavioral intention, or an emotion. In the context of the interview, this means that the signals sent by interviewers are an effective means for recruitment when, first, interviewers send signals to achieve their intended outcomes, second, applicants receive these signals, and third, applicants react in the desired way. Based on the signaling timeline model by Connelly et al. (2011), we suggest that a better understanding of the effects of interviewer IM can be achieved by differentiating the signals sent (interviewer IM), the signals received (perceived interviewer IM), and the reaction to the signals (recruiting outcomes).

### Interviewer impression management: what are the signals that are sent by interviewers?

While earlier recruitment research suggested that interviewers play only a minor role in influencing recruiting outcomes (e.g., Powell, 1984; Taylor and Bergmann, 1987; Rynes and Barber, 1990), recent work shows that interviewers actually play an important role in attracting and retaining applicants (e.g., Chapman et al., 2005; Carless and Imber, 2007; Wilhelmy et al., 2016). In addition, interviewers are well-aware of their role as representatives of the organization and of the emotional reactions they can elicit from applicants. In a qualitative study, for example, Wilhelmy et al. (2016) found that interviewers can have a multitude of goals, but that interviewers' primary concern was about representing the organization. Most interviewers reported that they tried to signal an attractive image of the organization to applicants to influence applicants' acceptance intention and organizational reputation, for example. Furthermore, in that study, interviewers' secondary concern centered on their personal interaction with applicants. Most interviewers reported that they tried to signal closeness to applicants, for example, to make applicants feel comfortable and elicit positive emotions in applicants.

To achieve their goals, interviewers can send signals to applicants in the form of verbal IM, that is, interviewers use the content of what they are saying to influence applicants' impressions. Furthermore, the IM literature makes a fundamental distinction between self-focused IM and other-focused IM (Tsai and Huang, 2014). From the perspective of the interviewer, a central self-focused IM behavior is organization-enhancement, sometimes used interchangeably with the term enhancement of the organization or self-enhancement. Organization-enhancement refers to stressing the positive qualities that one's organization possesses (e.g., pointing out strengths of the organization, Stevens et al., 1990; Wilhelmy et al., 2016). A central other-focused IM behavior of interviewers is applicant-enhancement, sometimes used interchangeably with the term ingratiation or other-enhancement, which refers to flattering the applicant (e.g., acknowledging past accomplishments of the applicant, Stevens et al., 1990; Wilhelmy et al., 2016).

In the present study, we chose to focus on these two interviewer IM behaviors because research indicates that they are relevant for applicants' positive attitudes, intentions, and emotions. Specifically, we assume that organization-enhancement is related to the goal of representing the organization such as creating an attractive image, whereas applicant-enhancement is related to the goal of putting applicants at ease such as creating a close relation image. For example, qualitative interviews with applicants revealed that applicants were impressed by interviewers who knew how to pitch the job and the organization and felt enthusiastic when interviewers encouraged them during the interview (Rynes et al., 1991). Similarly, Wilhelmy et al. (2016) found that interviewers from the health services field such as hospitals put emphasis on selling their organization by pointing out the advantages of the job and their respective hospital. In addition, in that study, it was found that interviewers reported trying to ensure that applicants would leave the interview room feeling good about themselves by paying attention to what applicants were saying and encouraging them during the interview. Furthermore, a laboratory experiment conducted by Stevens et al. (1990) found initial evidence that both organization-enhancement and applicant-enhancement elicited overall positive reactions in applicants.

### Perceived interviewer impression management: are the signal that are sent actually received by applicants?

Although research has shown that interviewers use IM to influence applicant impressions, an important question that remains is whether applicants discern interviewers' IM. On the one hand, the selection interview is a highly stressful event that can trigger strong emotions such as anxiety (McCarthy and Goffin, 2004). In contrast to interviewers, who are known to be responsive to applicants' IM, applicants are often nervous and anxious, which might not only lower their performance but also their attention span and the cognitive capacity to receive all the signals sent by interviewers (Tsai and Huang, 2014). On the other hand, signaling theory (Spence, 1973; Connelly et al., 2011; Bangerter et al., 2012) proposes that interviewers are an important source of information for applicants because applicants lack information about the job and the organization. As a consequence, applicants should try to pay attention to interviewers' behavior and to every piece of information that interviewers provide.

In line with the latter suggestion, previous studies indicated that applicants often perceive interviewer IM behavior. Wilhelmy et al. (2016) found that applicants were able to report examples of verbal interviewer IM behaviors that they had perceived in prior selection interviews. Thus, applicants seem to notice these behaviors. In addition, in Stevens et al.'s (1990) laboratory study, recruiters who praised the applicant were perceived as putting more persuasive effort into the interview compared to recruiters who used other IM behaviors. Furthermore, in the same study, participants reported a high degree of flattery when they were exposed to a recruiter who used applicant-enhancement. Therefore, given applicants' ability to report and comment on interviewer IM behavior (Stevens et al., 1990; Wilhelmy et al., 2016), and rooted in Connelly et al.'s (2011) signaling timeline model, we expected that organization-enhancement and applicant-enhancement used by interviewers during the interview would be perceived by applicants (see Figure 1).

*Hypothesis 1:* Interviewers' organization-enhancement in the interview will be positively related to applicants' subsequent perceptions of organization-enhancement.
*Hypothesis 2:* Interviewers' applicant-enhancement in the interview will be positively related to applicants' subsequent perceptions of applicant-enhancement.

### Recruiting outcomes: how do applicants react to the signals they receive?

The signaling timeline model by Connelly et al. (2011) suggests that when a signal is received, the receiver interprets the signal and reacts to it. In the context of recruitment, these reactions come in the form of recruiting outcomes, that is, applicants construe interviewer behavior as signals of how interesting a job and an organization is for them and also emotionally react to these signals (Rynes et al., 1991). Prior research indicates that important recruiting outcomes encompass applicant attitudes such as organizational prestige, which refers to the degree to which an organization is perceived as being well-regarded and reputable (Highhouse et al., 2003), applicant intentions such as offer acceptance intentions, which captures applicants' willingness to accept an offer for a job or a place at a university (Chapman et al., 2005), and applicant emotional reactions such as positive affect, which reflects applicants' positive emotional state (Watson et al., 1988), and interview self-efficacy, which captures the extent of their belief in their ability to succeed in an interview (Bauer et al., 1998).

In addition, previous work indicates that organization-enhancement plays a central role for applicants' attitudes and intentions, whereas applicant-enhancement is more important for applicants' emotions. Qualitative findings suggest that organization-enhancement is primarily used with the intention to create an attractive image to “sell” the organization to the applicant. By engaging in organization-enhancement, the interviewer attempts to quickly recruit applicants, enhance the organizations' reputation, and increase applicants' intention to accept an offer (Wilhelmy et al., 2016). Furthermore, in Stevens et al.'s (1990) laboratory study, participants watched three videos of interviewers, each of whom applied a different IM behavior to describe a hypothetical study program: self-promotion, which is in line with the conceptualization of organization-enhancement in the present study, other-enhancement, which is in line with the conceptualization of applicant-enhancement in the present study, and opinion-conformity, which combined aspects of organization-enhancement such as advantages of the study program and aspects of applicant-enhancement such as emphasizing the applicants' qualifications. The authors found that organization-enhancement was perceived as persuasive by some participants. However, the authors did not find full support for their assumption that compared to other IM behaviors, organization-enhancement would be most effective in making participants choose the hypothetical study program, particularly when organization-enhancement was used early in the interview. The authors interpreted this finding by suggesting that applicants may not always take organization-enhancement seriously or may not always pay attention to this kind of information. However, an important limitation of Stevens et al.'s (1990) study is that it did not consider whether applicants actually perceived the IM behavior that was presented in the videos. Therefore, given that organization-enhancement has been linked to interviewers' intention to create an attractive image (Stevens et al., 1990; Wilhelmy et al., 2016), we expect that perceived organization-enhancement would increase organizational prestige and applicants' intention to accept a potential offer from the organization.

*Hypothesis 3:* Applicants' perceptions of organization-enhancement will be positively related to (a) organizational prestige and (b) offer acceptance intentions after the interview.

Given the positive association that can be expected between the degree of IM sent by interviewers and the degree to which applicants perceive interviewer IM, and based on Connelly et al.'s (2011) model, we also aimed to test the whole signaling timeline mechanism for organization-enhancement. Extending the arguments above, we assume that interviewers' organization-enhancement during the interview has an indirect effect on organizational prestige and applicants' intention to accept a potential offer through perceived organization-enhancement (see Figure 1).

*Hypothesis 4:* Interviewers' organization-enhancement will be positively related to applicants' perceptions of organization-enhancement, which, in turn, will be positively related to (a) organizational prestige and (b) offer acceptance intentions after the interview.

Although applicants' attitudes and intentions are central to an organizations' success in recruiting new employees, researchers have recognized that applicants become emotionally invested. Thus, applicant emotions play a central role in the recruitment process (Truxillo et al., in press). This is particularly true for job interviews, which, unlike selection tests, involve a social exchange and can therefore trigger emotional reactions (McCarthy and Goffin, 2004).

So far, applicant reactions research has mainly focused on negative affective reactions such as interview anxiety (e.g., McCarthy and Goffin, 2004) or perceived strain (e.g., Merkulova et al., 2014). Several researchers, however, have called for research that considers applicants' affective reactions from another point of view, that of positive emotional reactions such as positive affect and self-efficacy (McCarthy et al., 2017; Truxillo et al., in press). In a recent framework on applicant perspectives in selection, McCarthy et al. (2017) emphasize the central role of applicant affect. They propose that treating applicants favorably during selection procedures enhances applicants' cognitive processing and triggers positive emotional reactions like positive affect. Specifically, applicants may feel more comfortable and self-confident when recruiters and interviewers show interest in their past achievements (Truxillo and Bauer, 2011). In this sense, perceived applicant-enhancement, which includes showing interest in applicants' past achievements, flatters, and reassures applicants, which makes them feel inspired and excited, and strengthens their beliefs in their own interview ability.

Despite the theoretical indications for a positive relationship between interviewers' applicant-enhancement and applicants' interview self-efficacy and positive affect, empirical evidence remains scarce. Regarding general self-efficacy, meta-analytic findings show that recruiters' attentiveness and friendly interpersonal treatment is related to applicants' self-perceptions such as self-efficacy after the selection process (Hausknecht et al., 2004). In addition, Stevens et al.'s (1990) study provided evidence that applicant-enhancement can be perceived as “buttering the ego” (p. 1087) by some applicants, but can also make applicants feel desired and reassured.

Regarding positive affect, Wilhelmy et al. (2016) found indications suggesting that applicant-focused IM is used with the aim of creating a close relation image and making applicants feel good in terms of creating a positive affective state. For example, interviewers compliment applicants with the intention to cheer them up. In addition, Stevens et al. (1990) proposed that in contrast to organization-enhancement, applicant-enhancement should provide weak information about prestige and suitability of the organization, but should provide a stronger basis for positive emotional reactions. In line with this assumption, they found that compared to other IM behaviors, applicant-enhancement had a positive influence on applicants' ratings of recruiter likeability, but no other affective reactions were assessed in the study. In addition, the study was limited to comparative effects of IM behaviors, that is, there was no variation in the degree of applicant-enhancement so that no main effect of applicant-enhancement on recruiting outcomes was assessed.

Given preliminary evidence regarding the positive link between perceived applicant-enhancement and feelings of reassurance and self-efficacy (Stevens et al., 1990; Hausknecht et al., 2004), along with interviewers' aim of creating a close relation image (Wilhelmy et al., 2016), and applicants' favorable affective reactions to applicant-enhancement (Stevens et al., 1990), we expected that perceived applicant-enhancement would increase applicants' positive affect and interview self-efficacy:

*Hypothesis 5:* Applicants' perceptions of applicant-enhancement will be positively related (a) to their positive affect and (b) to their interview self-efficacy after the interview.

Again, given the positive association that can be expected between the degree of IM sent by interviewers and the degree perceived by applicants based on Connelly et al.'s (2011) model, we also wanted to test the whole signaling timeline mechanism for applicant-enhancement. Extending the arguments above, we assume that interviewers' applicant-enhancement during the interview has an indirect effect on applicants' positive affect and interview self-efficacy through perceived applicant-enhancement (see Figure 1).

*Hypothesis 6:* Interviewers' applicant-enhancement will be positively related to applicants' perceptions of applicant-enhancement, which, in turn, will be positively related (a) to their positive affect and (b) to their interview self-efficacy after the interview.

## Methods

### Participants and procedures

#### Sample

Data for this field study were collected from 153 applicants who applied for a selective Bachelor's study program in organizational psychology at a university in Switzerland. For this program, applicants are selected in one of three interview periods a year that each last 2 weeks. The study sample included applicants from all three periods (November 2011, February 2012, and April 2012). Applicants were markedly older than high school graduates because this study program targets people with at least 1 year of prior work experience. Applicants' age ranged from 19 to 48 (*M* = 25.0, *SD* = 6.2), and their average work experience was 6.2 years (*SD* = 5.9). Of the 153 applicants, 74% were female. On average, they had participated in 5.0 interviews prior to this selection process (*SD* = 5.3). Of the 153 applicants, 90 received an offer by the university. This study was carried out with written informed consent from all participants.

This sample was appropriate for our study for several reasons: (1) The selection process for this study program was solely based on selection interviews (i.e., no other selection tools or admission tests were used), which enabled us to isolate the effects of interviewer IM without any confounding influences of other selection procedures, (2) there was competition between this university and others who offer similar study programs, which is an important prerequisite for some IM behaviors (please see Wilhelmy et al., 2016), (3) students had to pay very low tuition fees which makes the setting more similar to an application to a company.

#### Interviews

Interviews had a selection focus, were fairly structured, and based on interview guides that consisted of six topical areas (see Appendix A in Supplementary Material). For each topical area there were two to five obligatory questions containing both past behavior and situational questions. In other words, the amount and kind of questions asked did not vary across interviews. In addition, interviewer appearance was consistent across interviews (i.e., professional clothing such as button-down shirts and blazers).

Interviewers were not instructed to use IM, but they were in a position where they could use IM. For example, interviewers had enough latitude to use IM behaviors because they were free in how to ask the interview questions and whether to add any other information and personal chit-chat. Furthermore, interviewers were aware of the fact that other universities offered similar study programs and that the university wanted to retain its popularity. All of the 153 interviews were videotaped. On average, the interviews were 40.0 min long (*SD* = 7.0).

#### Interviewer teams

The unit of analysis of the present study was the individual interview because we were interested in the signals to which applicants were exposed to over the course of one interview. Each of the interviews was conducted with a different applicant by a team of two interviewers out of a pool of 17 interviewers. Interviewers alternated in asking the interview questions. Overall, there were 23 different interviewer teams. Interviewers were assigned to the interview dates based on their availability. All of the interviewers were well-trained. They had participated in an interviewer training by the university for which they were conducting the interviews. Furthermore, 12 of the interviewers had participated in additional interviewer training by other organizations or during their postgraduate training.

Eight of the interviewers were female (47%) and interviewer age ranged from 28 to 67 years (*M* = 40.7, *SD* = 10.8). All of the interviewers had an academic degree and were actively involved in the study program (13 of them as lecturers, 3 as Bachelor thesis advisors, and 1 as an examination committee member). On average, they had been working at the university for 5.9 years (*SD* = 7.2). The interviewers were diverse regarding their interview experience, which ranged from less than a year to 27 years (*M* = 5.6, *SD* = 7.5), and had conducted an average number of 102 interviews in their lives (*SD* = 134.0).

### Coding of interviewer IM behaviors

Unlike prior studies on interviewer characteristics and behaviors, we wanted to directly observe interviewer IM behaviors in a real selection setting by videotaping interviews and behaviorally coding these videotapes. Our approach to code IM behaviors was in line with previous studies that focused on applicants' IM (Stevens and Kristof, 1995; McFarland et al., 2005; Peeters and Lievens, 2006). Specifically, six I/O psychology graduate students served as coders (two male and four females; mean age = 24.7 years, *SD* = 1.5 years). They had gone through 5-h of frame-of-reference (FOR) training (Bernardin and Buckley, 1981) to recognize and record the frequency of interviewer IM behaviors. To record these behaviors, they used the INTERACT video coding software, which is a software to assess the frequency of behaviors by marking video sections with pre-assigned codes (Version 9, Mangold, 2010). Using this software, the amount of interviewer IM behaviors per interview was assessed based on the number of specific keystrokes: Coders watched the video recording of an interview and any time they identified an IM behavior, they pressed a key that was programmed to represent the specific interviewer IM behavior, respectively.

During the FOR training, coders were provided with definitions and examples of IM behaviors. Interviewers' organization-enhancement included statements promoting the university and the study program (e.g., “We have partner universities all over the world, you know”). Interviewers' applicant-enhancement included statements flattering and encouraging applicants (e.g., “that's impressive,” “very interesting,” “nice”). In addition, we also coded for defensive IM behaviors, which included interviewers' justifications and apologies (e.g., “sorry for making you wait”). However, these codes were neither included in the conceptual model nor in the data analyses^1^. As part of the FOR training, several video sections (overall 25 min) that each focused on different IM behaviors were coded independently by each coder to practice how to recognize and record each IM behavior. Based on these frequency-codings, coders received feedback. Any coding discrepancies were discussed to enhance coders' understanding of the IM behavior categories.

After the FOR training, ten 10-min video sections representing different sections of the interview (e.g., opening sentences, interview questions, closing sentences) were frequency-coded independently by each coder to evaluate the reliability of the codings. The overall percentage of agreement was found to be good (0.81 for organization-enhancement, 0.72 for applicant-enhancement, and 0.71 for defensive IM). The median of the interrater agreement of different pairs of coders was reasonable (κ = 0.66) considering that organization-enhancement and defensive IM occurred rather seldom (see Landis and Koch, 1977). Furthermore, the level of interrater agreement found in the present research is comparable to previous studies on interviewees' IM (e.g., Stevens and Kristof, 1995; McFarland et al., 2005).

Afterwards, the actual coding took place. As agreement between the coders was shown to be good, each video of the 153 selection interviews was coded by one coder. The frequency of organization-enhancement, applicant-enhancement, and defensive IM per video were assessed as the sum of the IM behaviors of the two interviewers because overall, that was what applicants were exposed to during the interview. We then calculated the relative frequencies of IM behaviors (i.e., IM behavior use divided by interview duration in minutes) to control for interview duration.

### Survey measures

Applicants were asked to complete two surveys at two different points during the interview process. The first survey (Time 1) was mailed to applicants 1–2 weeks prior to the interview along with an informed consent form and a cover letter. This survey also assessed demographic information such as age, gender, and years of prior work experience. The interviews were video recorded and directly after the interview, a second survey was handed to the applicants (Time 2).

Unless noted otherwise, five-point rating scales ranging from 1 = *strongly disagree* to 5 = *strongly agree* were used in this study. The items from the different scales used in the final model of this study are listed in Appendix B in Supplementary Material. Table 1 presents internal consistency coefficients for the measures for which they are applicable. All scales had adequate reliabilities.

**Table 1:** **Means, standard deviations, internal consistencies, and zero order correlations**.

**Variable**	***M***	***SD***	**1**	**2**	**3**	**4**	**5**	**6**	**7**	**8**	**9**	**10**	**11**	**12**	**13**	**14**	**15**	**16**
**DEMOGRAPHIC VARIABLES (T1)**																		
1. Gender (female = 0, male = 1)	0.25	0.44	–															
2. Age	25.07	6.18	−0.04	–														
3. Work experience	6.19	5.95	−0.04	0.84^**^	–													
**CONTROL VARIABLES (T1)**																		
4. Interview experience	5.07	5.30	0.00	0.11	0.06	–												
5. Organizational prestige	4.23	0.53	−0.03	−0.09	0.01	0.12	(0.77)											
6. Acceptance intention	10.48	1.15	0.01	−0.16^*^	−0.14	0.08	0.34^**^	–										
7. Positive affect	3.92	0.58	0.01	0.07	0.03	−0.09	0.26^**^	0.06	(0.72)									
8. Interview self-efficacy	4.03	0.63	0.04	0.12	0.14	0.11	0.20^*^	0.05	0.17^*^	(0.75)								
**INTERVIEWER IM (VIDEO CODED)**																		
9. Interviewers' organization-enhancement	0.13	0.14	−0.11	0.04	0.10	−0.01	0.02	0.02	−0.01	0.14	–							
10. Interviewers' applicant-enhancement	0.66	0.60	−0.06	−0.11	−0.03	0.03	0.07	0.13	0.08	0.13	0.13	–						
**PERCEIVED INTERVIEWER IM (T2)**																		
11. Perceived organization-enhancement	3.41	1.05	−0.09	0.03	−0.03	0.11	0.24^**^	0.14	0.11	0.05	0.18^*^	0.09	(0.82)					
12. Perceived applicant-enhancement	1.69	0.83	0.17^*^	−0.13	−0.10	0.03	0.13	0.18^*^	0.08	0.02	0.01	0.27^**^	0.32^**^	(0.86)				
**RECRUITING OUTCOMES (T2)**																		
13. Organizational prestige	4.30	0.59	−0.03	0.05	0.06	0.18^*^	0.68^**^	0.24^**^	0.25^**^	0.33^**^	0.08	0.07	0.29^**^	0.06	(0.85)			
14. Acceptance intention	10.21	1.53	−0.12	0.01	0.04	0.05	0.29^**^	0.70^**^	0.08	0.10	−0.01	0.05	0.17^*^	0.06	0.27^***^	–		
15. Positive affect	3.62	0.80	−0.02	0.01	0.04	−0.02	0.12	−0.02	0.31^**^	0.19^*^	−0.02	0.15	0.23^**^	0.13	0.19^*^	0.05	(0.82)	
16. Interview self-efficacy	3.48	0.80	0.13	0.11	0.14	0.20^*^	0.13	0.04	0.18^*^	0.50^**^	0.09	0.10	0.23^**^	0.18^*^	0.15	0.07	0.43^**^	(0.78)

*N = 153. T1 = Time 1, pre-interview applicant survey; T2 = Time 2, post-interview applicant survey; Video coded = Trained raters coded IM behavior that interviewers used during the selection interviews on the basis of video recordings of the interviews. Work experience was measured in years. Interview experience indicates the number of interviews applicants had before this interview. Acceptance intention was measured on an 11-point scale. The interviewer IM variables assessed through video coding indicate the number of times per minute that the IM behavior occurred during the interview. All other variables were measured on a 5-point scale. When appropriate, Cronbach's alpha values are shown on the diagonal in parentheses*.

* p < 0.05;

** p < 0.01;

*** *p < 0.001 (two-tailed)*.

#### Perceived organization-enhancement

To measure applicants' perceptions of interviewers' organization-enhancement at Time 2, we selected two items that were particularly appropriate from Turban and Dougherty (1992) and one item from Liden and Parsons (1986). An example item is “The interviewers attempted to present the study program in a positive way.” The internal consistency of this scale's ratings was good, with a coefficient alpha of 0.82.

#### Perceived applicant-enhancement

To measure applicants' perceptions of interviewers' applicant enhancement at Time 2, we selected two items that were particularly appropriate from Harn and Thornton (1985) and supplemented these by generating one item to increase reliability. An example item is “The interviewers complimented me.” The internal consistency of this scale's ratings was good, with a coefficient alpha of 0.86.

#### Organizational prestige

Organizational prestige was measured at Time 1 and Time 2 with four items from a scale developed by Highhouse et al. (2003) that was adapted to the context of a university. An example item is “Students are probably proud to say they study at this university.” The internal consistency of this scale's ratings was 0.77 for Time 1 and 0.85 for Time 2.

#### Acceptance intention

Applicants' acceptance intention was measured at Time 1 and Time 2 with a single item adapted from Powell and Goulet (1996). This measure has been widely used in previous studies (e.g., Chapman et al., 2003; Slaughter et al., 2004). Participants were asked to indicate “How likely are you to accept an offer from this university based on the information you have so far?” on an 11-point scale ranging from 1 = 0% to 11 = 100%.

#### Positive affect

Applicants' positive affect was measured at Time 1 and Time 2 with five items from Thompson's (2007) short-form of the Positive and Negative Affect Schedule (PANAS, Watson et al., 1988). Participants were asked to indicate to what extent each of the items described how they felt at the moment they completed the survey using a five-point scale ranging from 1 = *not at all* to 5 = *extremely*. An example item is “At the moment, I'm feeling active.” The internal consistency for this scale's ratings was 0.72 for Time 1 and 0.82 for Time 2.

#### Interview self-efficacy

Applicants' interview self-efficacy was measured at Time 1 and Time 2 with two items that were particularly appropriate from Horvath et al.'s (2000) self-efficacy measure and one item from Bauer et al.'s (1998) test-taking self-efficacy measure. We adapted the items to fit the context of an interview. An example item is “I believe I can perform well in interviews.” The internal consistency for this scale's ratings was 0.75 for Time 1 and 0.78 for Time 2.

#### Perceived interviewer competence

Applicants' perceptions of interviewer competence were measured at Time 2, but were not included in data analyses^2^. Perceived interviewer competence was measured with four items from Ridge and Reber (2002), Harris and Fink (1987), Carless and Imber (2007), and Turban and Dougherty (1992). Internal consistency of this scale's ratings was satisfying with a coefficient alpha of 0.74.

#### Control variables

As shown in Figure 1, several control variables were included based on theoretical justifications (Becker, 2005). We included applicants' prior interview experience as a control variable because some correlational evidence suggests that applicants with less interview experience attend to different aspects of interviewers' presentation than do applicants with more interview experience (Harris and Fink, 1987; Schreurs et al., 2005). Applicants interview experience was measured with an item developed by Harris and Fink (1987). Applicants were asked to indicate “How many prior interviews have you had in your life?” Because applicants' perceptions of interviewer IM and their reactions may depend on their prior interview experience, we included interview experience into our model as a control variable for both mediators and outcomes.

In addition, we measured pre-interview baseline values of our recruiting outcome variables at Time 1 and included them as control variables to acknowledge the fact that applicants differ in their attitudes, intentions, and emotions before the interview. To avoid psychometric problems that are associated with difference scores (see Edwards and Parry, 1993), we followed the recommendation for mediation designs to include baseline measures of the outcomes as control variables (MacKinnon et al., 2012). Specifically, we controlled each recruiting outcome for its respective baseline.

## Results

### Preliminary analyses and analytical approach

Table 1 presents descriptive statistics of the variables used in this study. To test our hypotheses in a methodologically rigorous manner and in one analytical model, we applied structural equation modeling (SEM). We analyzed the data using the statistical environment R (Version 3.3.2, R Development Core Team, 2016) and Mplus (Version 7.4, Muthén and Muthén, 2015). Given that applicants were nested in interviewer teams, we first explored the degree of potential non-independence of the observations. We examined the variances in IM behavior within and between interviewer teams by inspecting the intraclass correlations coefficients (ICCs). These indicated substantive dependency of interviewers' organization-enhancement on the interviewer teams, ICC(1) = 0.28, ICC(2) = 0.71, as well as a comparable dependency of interviewers' applicant-enhancement on the interviewer teams, ICC(1) = 0.26, ICC(2) = 0.69. In other words, ~30% of the variance in the IM behaviors that interviewers used was dependent on the interviewer team. Because this dependency is not taken into account by classical SEM (e.g., Heck and Thomas, 2015; Kline, 2016), we used the COMPLEX procedure and the MLR estimator in Mplus (Muthén and Satorra, 1995; Muthén and Muthén, 2015). This procedure corrects the χ^2^-test of model fit, the resulting fit indices, and the standard errors for non-independence of observations to ultimately deliver unbiased parameter estimates.

In our study, some variables could only be measured with single indicators. Specifically, interviewers' organization-enhancement and applicant-enhancement were each measured as cumulative frequencies (i.e., number of IM behavior coded per interview), interview experience was measured as the number of prior interviews that applicants have had, and acceptance intention was measured as applicants' self-reported likelihood to accept an offer from the university. Given that the residuals of single indicators cannot be estimated in SEM and thus potential measurement error of these variables cannot be taken into account, we followed recommendations by Hayduk and Littvay (2012) and estimated the residual variance for the single indicators (see also Little, 2013; Kline, 2016). By doing so, we estimated that 34% of the total variance of the interviewer IM variables was residual variance and 25% of the total variance of the single-item-indicators of acceptance intention and interview experience was residual variance. In addition, in light of the rather complex analytical procedure, we tried to reduce model complexity whenever possible (see Landis et al., 2000). We therefore followed the *construct-to-item-balance* approach (Little et al., 2002; Williams and O'Boyle, 2008) and created two parcels for the organizational prestige as well as for the positive affect measures.

To establish our postulated measurement model, we conducted three confirmatory factor analyses (CFAs; see Table 2). Residuals were not allowed to covary because there was no theoretical reason to assume they would (Cortina et al., 2016). Following recommendations by Little (2013) regarding acceptable model fit, we evaluated our models in light of five fit statistics: (1) absolute test of fit, χ^2^, (2) CFI ≥ 0.90, (3) TLI ≥ 0.90, (4) RMSEA ≤ 0.05, (5) SRMR ≤ 0.05. First, we estimated a one factor CFA in which all variables of interest loaded onto a single factor. This CFA did not converge and was therefore considered to be a misfit to the data. Next, we estimated a four-factor model where the items of each category of variables (predictors, mediators, outcomes, and controls) loaded onto a corresponding category variable. This four-factor model converged but displayed inacceptable fit to the data. Ultimately, we estimated the postulated measurement model with 13 factors, that is, we specified all theoretically postulated variables as separate entities. This model displayed acceptable fit, $\chi_{\left(138\right)}^{2}$ = 191.93, *p* < 0.01, CFI = 0.96, TLI = 0.93, RMSEA = 0.05 [90% CI: 0.03 −0.07, *p* = 0.44], SRMR = 0.04. Therefore, this measurement model was well-suited to test the SEM in the next step.

**Table 2:** **Model comparison based on the three confirmatory factor analyses**.

**Model**	**χ^2^**	***df***	***p***	**CFI**	**TLI**	**RMSEA**	**CI 90%**		**SRMR**
							**Lower**	**Upper**	
1 factor		Model did not converge							
4 factors	2109.86	67	<0.001	0.00	−0.61	0.24	0.24	0.25	0.17
13 factors	191.93	138	<0.01	0.96	0.93	0.05	0.03	0.07	0.04

*N = 153. CFI = Comparative fit index; TLI = Tucker-Lewis index; RMSEA = Root mean square error of approximation; CI = Confidence interval; SRMR = Standardized root mean square residual. The 90% confidence interval specifically refers to the RMSEA and tests whether close fit of the model to the data (RMSEA ≤ 0.05) can be accepted*.

To test our hypotheses in a rigorous manner, we estimated the theoretically postulated structural model, which follows the one depicted in Figure 1, but also tested all potential direct as well as indirect effects. In addition, control variables^3^ were taken into account by including paths from applicants' prior interview experience to the mediators and the outcome variables, and by also including paths from the respective baseline value (Time 1) of an outcome variable to its respective outcome at Time 2. This structural model displayed acceptable fit and was thus considered to be a valid representation of the data, $\chi_{\left(117\right)}^{2}$ = 221.30, *p* < 0.01, CFI = 0.95, TLI = 0.93, RMSEA = 0.05 [90% CI: 0.03 −0.07, *p* = 0.44], SRMR = 0.06.

### Test of hypotheses

**Figure 2:**
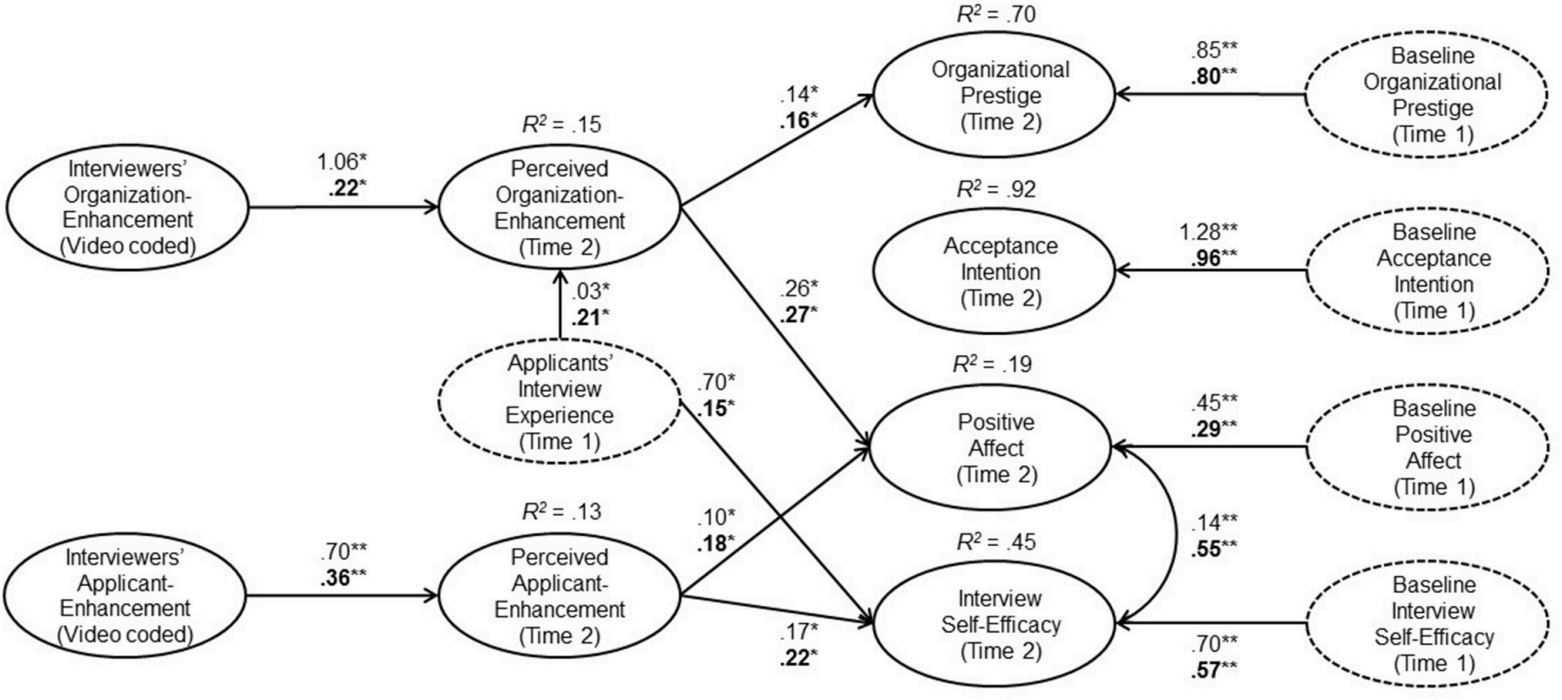
**Unstandardized and standardized (printed in bold) structural path coefficients of the final structural equation model**. Only significant paths are shown based on the unstandardized estimates. Dashed ellipses indicate control variables. Applicants' interview experience was used as a control variable for the mediators and recruiting outcomes. The baseline value (Time 1) for each recruiting outcome (Time 2) was used as a control variable. Path coefficients for control variables are presented in Table 3. ^*^p < 0.05; ^**^p < 0.01 (two-tailed).

Table 3 and Figure 2 provide unstandardized as well as standardized path coefficients of the structural equation model. Our first two hypotheses referred to the question of whether applicants would notice the IM behaviors that interviewers used during the interview. Consistent with Hypothesis 1, interviewers' organization-enhancement in the interview was positively related to applicants' subsequent perceptions of organization-enhancement, *b* = 1.06, *SE* = 0.51, *p* < 0.05. Furthermore, and in line with Hypothesis 2, interviewers' applicant-enhancement in the interview was positively related to applicants' subsequent perceptions of applicant-enhancement, *b* = 0.70, *SE* = 0.27, *p* < 0.05.

**Table 3:** **Structural path coefficients for mediators and outcome variables**.

	**Unstandardized estimate**	***SE***	**Standardized estimate**
**PERCEIVED ORGANIZATION-ENHANCEMENT (T2)**			
Interviewers' organization-enhancement (Video coded)	1.06^*^	0.51	0.22^*^
Interviewers' applicant-enhancement (Video coded)	0.22^†^	0.12	0.19^*^
Interview experience (T1)	0.03^*^	0.01	0.21^*^
**PERCEIVED APPLICANT-ENHANCEMENT (T2)**			
Interviewers' organization-enhancement (Video coded)	−0.47	0.99	−0.06
Interviewers' applicant-enhancement (Video coded)	0.70^*^	0.27	0.36^*^
Interview experience (T1)	0.01	0.03	0.03
**ORGANIZATIONAL PRESTIGE (T2)**			
Interviewers' organization-enhancement (Video coded)	0.19	0.35	0.04
Interviewers' applicant-enhancement (Video coded)	−0.05	0.10	−0.05
Perceived organization-enhancement (T2)	0.14^*^	0.06	0.16^*^
Perceived applicant-enhancement (T2)	−0.03	0.03	−0.05
Organizational prestige (T1)	0.85^*^	0.13	0.80^*^
Interview experience (T1)	0.01	0.01	0.08
**ACCEPTANCE INTENTION (T2)**			
Interviewers' organization-enhancement (Video coded)	−0.72	10.05	−0.06
Interviewers' applicant-enhancement (Video coded)	−0.27	0.29	−0.10
Perceived organization-enhancement (T2)	0.44^†^	0.23	0.18
Perceived applicant-enhancement (T2)	−0.11	0.09	−0.08
Acceptance intention (T1)	1.28^*^	0.20	0.96^*^
Interview experience (T1)	−0.02	0.02	−0.07
**POSITIVE AFFECT (T2)**			
Interviewers' organization-enhancement (Video coded)	−0.43	0.34	−0.09
Interviewers' applicant-enhancement (Video coded)	0.01	0.10	0.01
Perceived organization-enhancement (T2)	0.26^*^	0.13	0.27^*^
Perceived applicant-enhancement (T2)	0.10^*^	0.05	0.18^*^
Positive affect (T1)	0.45^*^	0.14	0.29^*^
Interview experience (T1)	−0.01^†^	0.01	−0.10
**INTERVIEW SELF-EFFICACY (T2)**			
Interviewers' organization-enhancement (Video coded)	0.14	0.52	0.02
Interviewers' applicant-enhancement (Video coded)	−0.10	0.16	−0.07
Perceived organization-enhancement (T2)	0.11	0.10	0.09
Perceived applicant-enhancement (T2)	0.17^*^	0.07	0.22^*^
Interview self-efficacy (T1)	0.70^*^	0.14	0.57^*^
Interview experience (T1)	0.02	0.02	0.15

*N = 153. Unstandardized and standardized parameter estimates stem from the final SEM. Estimates and standard errors account for clustering of applicants within interviewer teams, $\chi_{\left(117\right)}^{2}$ = 221.30, p < 0.01, CFI = 0.95, TLI = 0.93, RMSEA = 0.05 [90% CI:0.03–0.07, p = 0.44], SRMR = 0.06. SE = Standard error; T1 = Time 1, pre-interview applicant survey; T2 = Time 2, post-interview applicant survey; Video coded = Trained raters coded IM behavior that interviewers used during the selection interviews on the basis of video recordings of the interviews*.

† p < 0.10;

* *p < 0.05 (two-tailed)*.

In support of Hypotheses 3a, applicants' perceptions of organization-enhancement were positively related to organizational prestige, *b* = 0.14, *SE* = 0.06, *p* < 0.05. However, with regard to Hypothesis 3b, the association between applicants' perceptions of organization-enhancement and applicants' acceptance intention after the interview failed to reach significance at the conventional level (*p* = 0.06).

Hypothesis 4 specified a positive indirect effect of interviewers' organization-enhancement on (a) organizational prestige and (b) acceptance intentions through applicants' perceptions of organization-enhancement. Although the COMPLEX procedure of Mplus corrects the standard errors and consequently provides unbiased parameter estimates, it does not provide confidence intervals (Muthén and Muthén, 2015). Therefore, we applied the distribution-of-product method for building 95% confidence intervals for the indirect effects (MacKinnon et al., 2002) based on the corrected parameters using the R package *RMediation* (Version 1.1.4, Tofighi and MacKinnon, 2011). Indirect effects and confidence intervals are shown in Table 4. Consistent with Hypothesis 4a, the 95% confidence interval excluded zero and therefore indicated a significant positive indirect effect of interviewers' organization-enhancement via applicants' perceptions of organization-enhancement onto organizational prestige after the interview, *b* = 0.15, *SE* = 0.10, *p* < 0.05. With regard to Hypothesis 4b, the 95% confidence interval included zero, which means that the indirect effect of interviewers' organization-enhancement via applicants' perceptions of organization-enhancement onto acceptance intention after the interview was not significant.

**Table 4:** **Indirect effects and respective confidence intervals**.

**Indirect effect**	**Estimate**	***SE***	**CI 95%**	
			**Lower**	**Upper**
**ORGANIZATIONAL PRESTIGE**				
Organization-enhancement 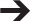 Perceived organization-enhancement 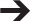 Organizational prestige	0.153^*^	0.098	0.002	0.379
Applicant-enhancement 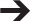 Perceived applicant-enhancement 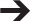 Organizational prestige	−0.017	0.026	−0.075	0.029
**ACCEPTANCE INTENTION**				
Organization-enhancement 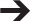 Perceived organization-enhancement 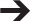 Acceptance intention	0.464	0.350	−0.051	1.288
Applicant-enhancement 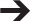 Perceived applicant-enhancement 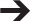 Acceptance intention	−0.075	0.071	−0.237	0.044
**POSITIVE AFFECT**				
Organization-enhancement 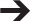 Perceived organization-enhancement 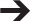 Positive affect	0.273^†^	0.197	−0.018	0.735
Applicant-enhancement 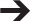 Perceived applicant-enhancement 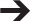 Positive affect	0.070^*^	0.043	0.003	0.168
**INTERVIEW SELF-EFFICACY**				
Organization-enhancement 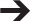 Perceived organization-enhancement 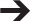 Interview self-efficacy	0.114	0.127	−0.091	0.412
Applicant-enhancement 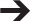 Perceived applicant-enhancement 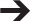 Interview self-efficacy	0.117^*^	0.069	0.010	0.274

*N = 153. Indirect effects are controlled for direct effects of Interviewer IM (video coded) on the outcome variables (Time 2) as well as baseline values of the outcome variables (Time 1). Confidence intervals were computed applying the distribution-of-product method based on the unstandardized parameters. SE, Standard error, CI, Confidence interval*.

† p < 0.10;

* *p < 0.05 (two-tailed)*.

Consistent with Hypotheses 5a, applicants' perceptions of applicant-enhancement were positively related to their positive affect, *b* = 0.10, *SE* = 0.05, *p* < 0.05. Furthermore, in support of Hypothesis 5b, applicants' perceptions of applicant-enhancement were positively related to their interview self-efficacy after the interview, *b* = 0.17, *SE* = 0.07, *p* < 0.05.

Finally, Hypothesis 6 specified a positive indirect effect of interviewers' applicant-enhancement on applicants' (a) positive affect and (b) their interview self-efficacy after the interview through their perceptions of applicant-enhancement. In line with Hypothesis 6a, the 95% confidence interval excluded zero and therefore indicated that the indirect effect of interviewers' applicant-enhancement through applicants' perceptions of applicant-enhancement was positively related to applicants' positive affect after the interview, *b* = 0.07, *SE* = 0.04, *p* < 0.05. Similarly, with regard to H6b, there was a positive indirect effect of interviewers' applicant-enhancement onto applicants' interview self-efficacy via applicants' perceptions of applicant-enhancement because the 95% confidence interval excluded zero, *b* = 0.12, *SE* = 0.07, *p* < 0.05.

## Discussion

Evidence from a growing number of studies suggests that the way interviewers are perceived by applicants can have tremendous effects on recruiting outcomes. However, this literature has yet to investigate how applicants respond to interviewer signals such as IM behavior. As a response to repeated calls for research on interviewer IM (e.g., Rosenfeld, 1997; Macan, 2009; Dipboye and Johnson, 2013) and, more specifically, on the mechanism by which applicants respond to interviewer IM (e.g., Gilmore et al., 1999; Celani and Singh, 2011; Tsai and Huang, 2014), our study offers a new perspective by not only incorporating IM behavior that interviewers apply, but also applicants' perceptions of interviewer IM and their reactions. Adopting a signaling perspective (Connelly et al., 2011), we conducted a field study to examine whether the signals that interviewers send (i.e., interviewer IM) are received by applicants (i.e., applicants' perceptions of interviewer IM), and how applicants react to the signals they receive (i.e., recruiting outcomes). We provide a conceptual model (Figure 1) that captures a signaling timeline of interviewer IM and delineates two interviewer goals (representing their organization and making the applicant feel good) that can each be achieved by using a different interviewer IM behavior (organization-enhancement and applicant-enhancement). Our study goes beyond the two sole hitherto published studies on interviewer IM (i.e., Stevens et al., 1990; Wilhelmy et al., 2016) by examining how applicants perceive and react to interviewer IM.

Our results yielded three key findings. First, we found that the signals that interviewers send are received by applicants. When interviewers enhanced the image of the organization to a stronger degree during the interview, applicants indeed reported more perceived organization-enhancement after the interview. Similarly, when interviewers praised and complimented the applicant to a stronger degree during the interview, applicants reported more applicant-enhancement after the interview. Second, we found that applicants react to the signals they receive. Applicants rated the organization's prestige after the interview more positively when they perceived interviewers to be engaging in more organization-enhancement behavior during the interview, and this was true even after controlling for perceptions of prestige before the interview. In addition, when interviewers were perceived to be engaging in more applicant-enhancing behaviors, applicants reported more positive affect and interview self-efficacy after the interview, even after controlling for initial affect and interview self-efficacy. Third, we found support for indirect effects delineated in our signaling timeline model for three of the four recruiting outcomes. We found an indirect effect of interviewers' organization-enhancement on organizational prestige through applicants' perceptions of organization-enhancement as well as indirect effects of interviewers' applicant-enhancement on applicants' positive affect and interview self-efficacy through applicants' perceptions of applicant-enhancement. To our knowledge, these results are the first to show that interviewer IM behaviors can influence recruiting outcomes through applicants' perceptions of these behaviors.

### Implications for theory and practice

This study has several theoretical and practical implications. Our study relates to signaling theory (Spence, 1973; Connelly et al., 2011; Bangerter et al., 2012) by proposing and testing a signaling timeline model to explain the effects of interviewer IM. It sheds light on the effects of interviewer IM by considering both the interviewer's perspective in terms of sending signals and the applicant's perspective in terms of receiving and responding to signals. Specifically, we found that for interviewer IM (i.e., signals sent) to have an effect on recruiting outcomes, applicants need to perceive interviewers' IM behavior (i.e., signals received), and respond to it (i.e., reactions to signals). This finding emphasizes that in the context of the interview, it is important to conceptually and empirically distinguish the signals sent from the signals received because to have an effect, signals that are sent by interviewers need to be received by applicants.

Furthermore, we expand previous theoretical frameworks on IM (e.g., Gilmore et al., 1999; Tsai and Huang, 2014) by providing evidence that different kinds of interviewer IM (organization-enhancement and applicant-enhancement) are conceptually related to different goals of interviewers (representing the organization and making applicants feel good): Organization-enhancement seems to be particularly effective at influencing applicants' positive attitudes toward the organization, whereas applicant-enhancement seems to be particularly effective at influencing applicants' positive emotions. This is in line with and further corroborates prior propositions that one kind of IM behavior may not be relevant for every purpose (Tsai and Huang, 2014; Wilhelmy et al., 2016). In addition, our findings stress that it is crucial to consider various recruiting outcomes, including those that reflect organizations' perspective such as organizational reputation and prestige, but also criteria that reflect applicants' perspective such as their positive affect and belief in the ability to succeed with an interview.

The findings of the present study do not only benefit the research community but also have implications for practitioners. Our finding that interviewers' organization-enhancement and applicant-enhancement can influence recruiting outcomes provides indications on the opportunity of increasing organizations' competitive advantage through signals that are sent to applicants in the course of the interview process. For instance, organizations could conduct training sessions to enhance interviewers' IM skills. Specifically, organization-enhancement could help interviewers highlight strengths of the organization and attract the best applicants. Applicant-enhancement, in contrast, could help interviewers to make applicants feel wanted and thus foster positive emotions and feelings of self-confidence, which is, for example, important for word-of-mouth recommendations. Nonetheless, the effect sizes in the present study were relatively small, which potentially limits the practical relevance of the findings. The small effect sizes, however, could also be due to the large number of factors that influence recruiting outcomes. In addition, a phenomenon can be important despite small effect sizes (Cortina and Landis, 2009). We therefore believe that interviewers' IM has the potential to be effectively used by organizations, but the findings presented in this paper should be bolstered by insightful future research before more specific practical recommendations can be made.

Despite these potential benefits, interviewer IM may lead to conflicts between the selection and recruitment needs of employers. Interviewer IM may enhance the recruitment function of the interview but may adversely affect the psychometric qualities such as inter-rater reliability (because of deviations from standardization) and criterion-related validity (because of the introduction of systematic error) and thus impede the selection function. For example, Marr and Cable (2014) found that interviewers' selling-orientation reduced their judgment accuracy and the interview's predictive validity. However, as Tsai and Huang (2014) pointed out, this does not mean that employers have to sacrifice their recruitment needs for their selection needs. For example, in order to achieve a balance between the recruitment and selection functions, interviewers may consider conducting two separate interviews, “one designed strictly for prediction and the other designed to allow an informal question-and-answer session to meet the needs of applicants” (Kohn and Dipboye, 1998, p. 839). However, as separate interviews involve additional costs, another solution might be to conduct highly structured interviews but incorporate a more personal interview stage, for example at the beginning and the end of the interview (Schuler and Funke, 1989; Chapman and Rowe, 2001). In addition, some researchers and practitioners may argue against the use of interviewer IM because interviewers might exaggerate information and try to deceive applicants when applying IM behaviors. Unrealistic expectations can ultimately result in negative affective reactions on the part of employees (Wanous et al., 1992). Thus, interviewers should avoid pursuing the wrong applicants on the wrong terms. A practical solution may be to combine IM with realistic job previews (RJPs, Wanous, 1976) in terms of presenting positive attributes while also informing applicants about possible downsides of the job and the organization (see Wilhelmy et al., 2016).

### Limitations and implications for future research

Although results of the present study provide valuable insights into the effects of interviewer IM, the study is not without limitations. For example, our data was based on real selection interviews that were conducted as part of the selection process for one specific academic organization. This may call the generalizability of our study into question, but it does not diminish the relevance of this sample because the challenges that universities face regarding their recruitment efforts are similar to those in private enterprises in terms of competition with other universities and the need to balance selection and recruitment aims (e.g., Colarelli et al., 2012). Furthermore, it is important to note that even though we examined applicants who applied for a Bachelor's study program, our sample does not represent a student sample. In fact, in our study, applicants had work experience and were markedly older than high school graduates with an average age of 25 years. The reason for this is that the study program is specifically designed for individuals with prior work experience who want to complement their training and education with an additional Bachelor's degree.

Moreover, the present study was conducted in the field using pre-interview and post-interview applicant survey data and video coding of interviewers' IM behavior, but mediators were measured at the same time as the outcomes. This raises common method bias concerns. However, these concerns are alleviated to some extent by using two measurement points (before and after the interview) and two data sources (video recordings and applicant surveys) to separate the data collection on predictors and outcomes (following recommendations by Podsakoff et al., 2003) and to incorporate baselines of the outcome variables (following recommendations by MacKinnon et al., 2012). Nonetheless, future research should use a more rigorous research design with multiple measurement points to obtain predictors, mediators, and outcomes at different points in time to strengthen causal inferences (see Ployhart and MacKenzie, 2015).

A strength of the current study, which is also a potential limitation, is the fact that we focused on interviewer IM behavior that was actually applied during interviews and used an extensive behavioral video coding procedure to gain valuable insights into the frequency of these behaviors. Even though this methodology is in line with previous IM research (e.g., Stevens and Kristof, 1995; Ellis et al., 2002; McFarland et al., 2005; Peeters and Lievens, 2006), it remains unclear what intentions interviewers had when they used these IM behaviors. Future research should put more emphasis on interviewer IM intentions, for example, by combining video ratings as used in the present study with self-ratings of IM behavior (Levashina and Campion, 2007).

In the present study, interview structure, interview questions, and the relation of selection and recruitment goals were held constant across interviews. However, interview structure and the ratio of recruitment vs. selection goals may be important boundary conditions of interviewer IM and should therefore be studied. Specifically, researchers may focus on the possibility that there might be a minimum level of freedom regarding the interview content and a minimum level of recruitment objectives needed for interviewer IM to occur and to be effectively applied. Relatedly, the present study focused on the signals that were sent by a team of two interviewers who alternated in asking the interview questions. To understand how interviewer IM evolves in the first place, it is important to also consider the individual interviewer as the unit of analysis. It could be that some interviewers use the same kind and degree of IM in every interview and that others adapt their IM behavior to every interview situation and every applicant. Thus, we encourage future research to study larger samples of interviewers and examine the degree to which an interviewer's IM behavior remains stable across interviews (e.g., depending on interview guidelines of the organization and the interviewer's personality), and to what degree interviewers adapt their IM from interview to interview (e.g., depending on how much they want to win an applicant over).

The current study provides indications that interviewers' IM is indeed perceived by applicants, and that it can influence applicants' attitudes, intentions, and emotions. However, as a starting point for this line of research, we only focused on two central IM behaviors, namely organization-enhancement and applicant-enhancement. Qualitative findings revealed that interviewers use a broad range of different verbal IM behaviors that go beyond organization-enhancement and applicant-enhancement such as demonstrating humor and depreciating applicants (Wilhelmy et al., 2016). In addition, interviewers not only use verbal IM but also artifactual IM (i.e., using appearance and visual information to influence applicant impressions such as displaying application documents) and administrative IM (i.e., using timing of communication and providing services to influence applicants' impressions such as incorporating future colleagues). Future research should consider this broad range of interviewer IM behavior to provide more comprehensive insights into its effects.

In addition, future research should consider interactive effects and different combinations of interviewer IM. The current study examined the effects of organization-enhancement and applicant-enhancement on recruiting outcomes, but there could be different configurations of interviewer IM used by an interviewer team (e.g., mainly organization-enhancement vs. mainly applicant-enhancement vs. both organization-enhancement and applicant-enhancement vs. neither organization-enhancement nor applicant-enhancement), and these different configurations could have different effects on recruiting outcomes. Therefore, to examine interactive effects with increased statistical power to detect those effects, we encourage future research to use experimental study designs to specifically vary and combine interviewers' IM behaviors. In addition, in real selection settings with large samples of interviewers, the prevalence and effects of different configurations of interviewer IM could be examined through latent profile analyses. Furthermore, Stevens et al. (1990) found that organization- and applicant-enhancement can have a positive influence on recruitment by increasing applicants' perceptions of interviewer competence, but can also backfire by appearing presumptuous to applicants. This raises the question of what kind of IM may tip the scale between perceiving organization-enhancement as valuable information and as a signal of interviewer competence vs. arrogance. Future research exploring the interactive effects of different kinds of interviewer IM behavior and the role of perceived interviewer competence vs. arrogance is warranted.

Another potential limitation is that the current study examined the effects of interviewer IM on applicants' attitudes, intentions, and emotions, but not on behavioral outcomes. For example, we do not know the extent to which applicants' job choice decisions would be influenced by interviewer IM. Recent findings from a military context indicate that perceived recruiter characteristics such as personableness can influence applicants' acceptance decisions (Harold et al., 2016). However, as Tsai and Huang (2014) point out, it is possible that applicants' attitudes and intentions after the interview are influenced by interviewer IM, but that their actual job choice behavior will be primarily based on information provided by other sources than the interviewer such as friends or insiders that work at the organization. Future research should therefore employ longitudinal designs in selection settings where there is enough variance in applicants' job choice decisions to answer this important question.

Finally, several unexpected findings warrant attention. In the current study, applicants' perceptions of organization-enhancement were not significantly related to their intention to accept a potential offer. Stevens et al. (1990) found that only a few of the study participants opted for the university that was presented by a recruiter who used organization-enhancement, and that this was because the arguments presented were not seen as convincing. Thus, future research should consider how applicants interpret organization-enhancement, and how much weight this information carries for different applicants. Another interesting finding is that applicants' perceptions of organization-enhancement were related to applicants' positive affect after the interview. This may be because strengths and advantages of the study program and the organization that were pointed out by interviewers may increase applicants' excitement and enthusiasm for the interview and thus elicit positive affective reactions in applicants. Lastly, the mean level of the recruiting outcomes decreased after the interview. This is in line with prior studies: When comparing means before and after the interview, applicants' acceptance intention and ratings of organizational attractiveness were often found to decrease (Powell, 1991; Carless, 2005; Carless and Imber, 2007). In another study, applicants' test taking self-efficacy was found to decrease after the selection process (Bauer et al., 1998). A possible explanation for this decrease in recruiting outcomes is that after the interview, applicants are often overwhelmed and buried in information that they need to process. In addition, it could be that the interview helped applicants decide against the organization and select themselves out of the process, which might have decreased the mean level of recruiting outcomes. Furthermore, we do not know how much the mean level of the recruiting outcomes would have decreased without any interviewer IM during the interviews. Future research could therefore use longitudinal, quasi-experimental designs with several groups that differ in the amount of interviewer IM to examine main effects of interviewer IM on recruitment outcomes.

## Conclusion

The insights gained in this study do not only provide valuable implications for current interview practice and theory, but also lay the foundation for more multifaceted, insightful research on signaling processes in the future. Overall, our results suggest that interviewer IM behaviors can serve as effective means for recruitment through applicants' perceptions of interviewer IM. These effects should be tested across different organizations for different kinds of IM such as paraverbal and non-verbal IM, and by combining video ratings and self-report measures of interviewer IM. In addition, we encourage future research to further explore the conditions within organizations, interview settings, and interviewers that facilitate the use and effectiveness of interviewer IM. We hope that future research on the topics and questions that we have mentioned will provide further insight into how and when interviewer IM can lead to positive outcomes of the interview process for both applicants and organizations.

## Ethics statement

This study was carried out with written informed consent from all participants in accordance with the requirements of the German Association for Psychology.

## Author contributions

AW contributed substantially to the conception, design, acquisition, analysis, and interpretation of data for the work, drafted the work, revised the work critically for important intellectual content, approved the final version to be published, and agreed to be accountable for all aspects of the work in ensuring that questions related to the accuracy or integrity of any part of the work are appropriately investigated and resolved. MK and KM provided substantially to the conception, acquisition, interpretation of data, revised the work critically for important intellectual content, approved the final version to be published, and agreed to be accountable for all aspects of the work in ensuring that questions related to the accuracy or integrity of any part of the work are appropriately investigated and resolved. MG provided substantially to the analysis of data, revised the work critically for important intellectual content, approved the final version to be published, and agreed to be accountable for all aspects of the work in ensuring that questions related to the accuracy or integrity of any part of the work are appropriately investigated and resolved.

### Conflict of interest statement

The authors declare that the research was conducted in the absence of any commercial or financial relationships that could be construed as a potential conflict of interest.

## Footnotes

^1^Defensive IM behaviors were coded for the sake of completeness, but were not included in data analyses. Applicants usually comply with interviewers' control in the interview so that interviewers should feel no need to justify their behaviors (Kirkwood and Ralston, 1999). In line with this assumption, interviewers rarely engaged in defensive IM in the present study (*M* = 0.01 per minute, *SD* = 0.03). Thus, the main focus of this study was on organization-enhancement and applicant-enhancement.

^2^Applicants' perceptions of interviewer competence were measured, but due to a legitimate conceptual comment from the Editor in an earlier version of this manuscript, this variable was not included in further analyses.

^3^We repeated the SEM analysis without any control variables and the pattern of results remained the same.

## References

[B1] Bangerter A. Roulin N. König C. J. (2012). Personnel selection as a signaling game. J. Appl. Psychol. 97, 719–738. 10.1037/a0026078 22040263

[B2] Bauer T. N. Maertz C. P. Jr. Dolen M. R. Campion M. A. (1998). Longitudinal assessment of applicant reactions to employment testing and test outcome feedback. J. Appl. Psychol. 83, 892–903. 10.1037/0021-9010.83.6.892

[B3] Becker T. E. (2005). Potential problems in the statistical control of variables in organizational research: a qualitative analysis with recommendations. Organ. Res. Methods 8, 274–289. 10.1177/1094428105278021

[B4] Bernardin H. J. Buckley M. R. (1981). Strategies in rater training. Acad. Manage. Rev. 6, 205–212.

[B5] Carless S. A. (2005). Person-job fit versus person–organization fit as predictors of organizational attraction and job acceptance intentions: a longitudinal study. J. Occup. Organ. Psychol. 78, 411–429. 10.1348/096317905X25995

[B6] Carless S. A. Imber A. (2007). The influence of perceived interviewer and job and organizational characteristics on applicant attraction and job choice intentions: the role of applicant anxiety. Int. J. Select. Assess. 15, 359–371. 10.1111/j.1468-2389.2007.00395.x

[B7] Celani A. Singh P. (2011). Signaling theory and applicant attraction outcomes. Pers. Rev. 40, 222–238. 10.1108/00483481111106093

[B8] Chapman D. S. Rowe P. M. (2001). The impact of videoconference technology, interview structure, and interviewer gender on interviewer evaluations in the employment interview: a field experiment. J. Occup. Organ. Psychol. 74, 279–298. 10.1348/096317901167361

[B9] Chapman D. S. Uggerslev K. L. Carroll S. A. Piasentin K. A. Jones D. A. (2005). Applicant attraction to organizations and job choice: a meta-analytic review of the correlates of recruiting outcomes. J. Appl. Psychol. 90, 928–944. 10.1037/0021-9010.90.5.928 16162065

[B10] Chapman D. S. Uggerslev K. L. Webster J. (2003). Applicant reactions to face-to-face and technology-mediated interviews: a field investigation. J. Appl. Psychol. 88, 944–953. 10.1037/0021-9010.88.5.944 14516254

[B11] Colarelli S. M. Monnot M. J. Ronan G. F. Roscoe A. M. (2012). Administrative assumptions in top-down selection: a test in graduate school admission decisions. Appl. Psychol. 61, 498–512. 10.1111/j.1464-0597.2011.00480.x

[B12] Connelly B. L. Certo S. T. Ireland R. D. Reutzel C. R. (2011). Signaling theory: a review and assessment. J. Manage. 37, 39–67. 10.1177/0149206310388419

[B13] Cortina J. M. Green J. P. Keeler K. R. Vandenberg R. J. (2016). Degrees of freedom in SEM: are we testing the models that we claim to test? Organ. Res. Methods. [Epub ahead of print]. 10.1177/1094428116676345

[B14] Cortina J. M. Landis R. S. (2009). When small effect sizes tell a big story, and when large effect sizes don't, in Statistical and Methodological Myths and Urban Legends, eds Lance C. E. Vandenberg R. J. (New York, NY: Routledge), 287–308.

[B15] Dipboye R. L. Johnson S. K. (2013). Understanding and improving employee selection interviews, in APA Handbook of Testing and Assessment in Psychology, ed Geisinger K. F. (Washington, DC: American Psychological Association), 479–499.

[B16] Dipboye R. L. Macan T. H. Shahani-Denning C. (2012). The selection interview from the interviewer and applicant perspectives: can't have one without the other, in The Oxford Handbook of Personnel Assessment and Selection, ed Schmitt N. (New York, NY: Oxford University Press), 323–352.

[B17] Edwards J. R. Parry M. E. (1993). On the use of polynomial regression equations as an alternative to difference scores in organizational research. Acad. Manage. J. 36, 1577–1613. 10.2307/256822

[B18] Ellis A. P. West B. J. Ryan A. M. DeShon R. P. (2002). The use of impression management tactics in structured interviews: a function of question type? J. Appl. Psychol. 87, 1200–1208. 10.1037/0021-9010.87.6.1200 12558226

[B19] Farago B. Zide J. S. Shahani-Denning C. (2013). Selection interviews: role of interviewer warmth, interview structure, and interview outcome in applicants' perceptions of organizations. Consult. Psychol. J. Pract. Res. 65, 224–239. 10.1037/a0034300

[B20] Gilmore D. C. Stevens C. K. Harrel-Cook G. Ferris G. R. (1999). Impression management tactics, in The Employment Interview Handbook, eds Eder R. W. Harris M. M. (Thousand Oaks, CA: Sage), 321–336.

[B21] Harn T. J. Thornton G. C. (1985). Recruiter counselling behaviours and applicant impressions. J. Occup. Psychol. 58, 57–65. 10.1111/j.2044-8325.1985.tb00180.x

[B22] Harold C. M. Holtz B. C. Griepentrog B. K. Brewer L. M. Marsh S. M. (2016). Investigating the effects of applicant justice perceptions on job offer acceptance. Pers. Psychol. 69, 199–227. 10.1111/peps.12101

[B23] Harris M. M. Fink L. S. (1987). A field study of applicant reactions to employment opportunities: does the recruiter make a difference? Pers. Psychol. 40, 765–784. 10.1111/j.1744-6570.1987.tb00623.x

[B24] Hausknecht J. P. Day D. V. Thomas S. C. (2004). Applicant reactions to selection procedures: an updated model and meta-analysis. Pers. Psychol. 57, 639–683. 10.1111/j.1744-6570.2004.00003.x

[B25] Hayduk L. A. Littvay L. (2012). Should researchers use single indicators, best indicators, or multiple indicators in structural equation models? BMC Med. Res. Methodol. 12:159. 10.1186/1471-2288-12-159 23088287 PMC3506474

[B26] Heck R. H. Thomas S. L. (2015). An Introduction to Multilevel Modeling Techniques, 3rd Edn. New York, NY: Routledge.

[B27] Highhouse S. Lievens F. Sinar E. F. (2003). Measuring attraction to organizations. Educ. Psychol. Meas. 63, 986–1001. 10.1177/0013164403258403

[B28] Horvath M. Ryan A. M. Stierwalt S. L. (2000). The influence of explanations for selection test use, outcome favorability, and self-efficacy on test-taker perceptions. Organ. Behav. Hum. Decis. Process. 83, 310–330. 10.1006/obhd.2000.2911 11056073

[B29] Kirkwood W. G. Ralston S. M. (1999). Inviting meaningful applicant performances in employment interviews. J. Bus. Commun. 36, 55–76. 10.1177/002194369903600103

[B30] Kline R. B. (2016). Principles and Practice of Structural Equation Modeling, 4th Edn. New York, NY: Guilford Press.

[B31] Kohn L. S. Dipboye R. L. (1998). The effects of interview structure on recruiting outcomes. J. Appl. Soc. Psychol. 28, 821–843. 10.1111/j.1559-1816.1998.tb01733.x

[B32] Landis J. R. Koch G. G. (1977). The measurement of observer agreement for categorical data. Biometrics 33, 159–174. 10.2307/2529310 843571

[B33] Landis R. S. Beal D. J. Tesluk P. E. (2000). A comparison of approaches to forming composite measures in structural equation models. Organ. Res. Methods 3, 186–207. 10.1177/109442810032003

[B34] Levashina J. Campion M. A. (2007). Measuring faking in the employment interview: development and validation of an Interview Faking Behavior scale. J. Appl. Psychol. 92, 1638–1656. 10.1037/0021-9010.92.6.1638 18020802

[B35] Levashina J. Hartwell C. J. Morgeson F. P. Campion M. A. (2014). The structured employment interview: narrative and quantitative review of the research literature. Pers. Psychol. 67, 241–293. 10.1111/peps.12052

[B36] Liden R. C. Parsons C. K. (1986). A field study of job applicant interview perceptions, alternative opportunities, and demographic characteristics. Pers. Psychol. 39, 109–122. 10.1111/j.1744-6570.1986.tb00577.x

[B37] Little T. D. (2013). Longitudinal Structural Equation Modeling. New York, NY: Guilford Press.

[B38] Little T. D. Cunningham W. A. Shahar G. Widaman K. F. (2002). To parcel or not to parcel: exploring the question, weighing the merits. Struct. Equat. Model. 9, 151–173. 10.1207/S15328007SEM0902_1

[B39] Macan T. H. (2009). The employment interview: a review of current studies and directions for future research. Hum. Resour. Manage. Rev. 19, 203–218. 10.1016/j.hrmr.2009.03.006

[B40] MacKinnon D. P. Coxe S. Baraldi A. N. (2012). Guidelines for the investigation of mediating variables in business research. J. Bus. Psychol. 27, 1–14. 10.1007/s10869-011-9248-z 25237213 PMC4165346

[B41] MacKinnon D. P. Lockwood C. M. Hoffman J. M. West S. G. Sheets V. (2002). A comparison of methods to test mediation and other intervening variable effects. Psychol. Methods 7, 83–104. 10.1037/1082-989X.7.1.83 11928892 PMC2819363

[B42] Mangold (2010). INTERACT Quick Start Manual V2.4. Available online at: www.mangold-international.com

[B43] Marr J. C. Cable D. M. (2014). Do interviewers sell themselves short? The effects of selling orientation on interviewers' judgements. Acad. Manage. J. 57, 624–651. 10.5465/amj.2011.0504

[B44] McCarthy J. M. Goffin R. (2004). Measuring job interview anxiety: beyond weak knees and sweaty palms. Pers. Psychol. 57, 607–637. 10.1111/j.1744-6570.2004.00002.x

[B45] McCarthy J. M. Bauer T. N. Truxillo D. M. Anderson N. R. Costa A. C. Ahmed S. M. (2017). Applicant perspectives during selection: a review addressing “So what?,” “What's new?,” and “Where to next?” J. Manage. [Epub ahead of print]. 10.1177/0149206316681846

[B46] McFarland L. A. Yun G. Harold C. M. Viera L. Jr. Moore L. G. (2005). An examination of impression management use and effectiveness across assessment center exercises: the role of competency demands. Pers. Psychol. 58, 949–980. 10.1111/j.1744-6570.2005.00374.x

[B47] Merkulova N. Melchers K. G. Kleinmann M. Annen H. Szvircsev Tresch T. (2014). Effects of individual differences on applicant perceptions of an operational assessment center. Int. J. Select. Assess. 22, 355–370. 10.1111/ijsa.12083

[B48] Muthén B. O. Satorra A. (1995). Complex sample data in structural equation modeling. Sociol. Methodol. 25, 267–316. 10.2307/271070

[B49] Muthén L. K. Muthén B. O. (2015). Mplus User's Guide, 7th Edn. Los Angeles, CA: Muthén & Muthén.

[B50] Peeters H. Lievens F. (2006). Verbal and nonverbal impression management tactics in behavior description and situational interviews. Int. J. Select. Assess. 14, 206–222. 10.1111/j.1468-2389.2006.00348.x

[B51] Ployhart R. E. MacKenzie W. I. Jr. (2015). Two waves of measurement do not a longitudinal study make, in More Statistical and Methodological Myths and Urban Legends, eds Lance C. E. Vanderberg R. J. (New York, NY: Routledge), 85–99.

[B52] Podsakoff P. M. MacKenzie S. B. Lee J. Y. Podsakoff N. P. (2003). Common method biases in behavioral research: a critical review of the literature and recommended remedies. J. Appl. Psychol. 88, 879–903. 10.1037/0021-9010.88.5.879 14516251

[B53] Powell G. N. (1984). Effects of job attributes and recruiting practices on applicant decisions: a comparison. Pers. Psychol. 37, 721–732. 10.1111/j.1744-6570.1984.tb00536.x

[B54] Powell G. N. (1991). Applicant reactions to the initial employment interview: exploring theoretical and methodological issues. Pers. Psychol. 44, 67–83. 10.1111/j.1744-6570.1991.tb00691.x

[B55] Powell G. N. Goulet L. R. (1996). Recruiters' and applicants' reactions to campus interviews and employment decisions. Acad. Manage. J. 39, 1619–1640. 10.2307/257071

[B56] R Development Core Team (2016). R: A Language and Environment for Statistical Computing. Vienna.

[B57] Ridge R. D. Reber J. S. (2002). “I think she's attracted to me”: the effect of men's beliefs on women's behavior in a job interview scenario. Basic Appl. Soc. Psychol. 24, 1–14. 10.1207/S15324834BASP2401_1

[B58] Rosenfeld P. (1997). Impression management, fairness, and the employment interview. J. Bus. Ethics 16, 801–808. 10.1023/A:1017972627516

[B59] Rynes S. L. Barber A. E. (1990). Applicant attraction strategies: an organizational perspective. Acad. Manage. Rev. 15, 286–310.

[B60] Rynes S. L. Bretz R. D. Gerhart B. (1991). The importance of recruitment in job choice: a different way of looking. Pers. Psychol. 44, 487–521. 10.1111/j.1744-6570.1991.tb02402.x

[B61] Schlenker B. R. (1980). Impression Management: The Self-Concept, Social Identity, and Interpersonal Relations. Monterey, CA: Brooks-Cole.

[B62] Schreurs B. Derous E. De Witte K. Proost K. Andriessen M. Glabeke K. (2005). Attracting potential applicants to the military: the effects of initial face-to-face contacts. Hum. Perform. 18, 105 10.1207/s15327043hup1802_1

[B63] Schuler H. Funke U. (1989). The interview as a multimodal procedure, in The Employment Interview: Theory, Research, and Practice, eds Eder R. W. Ferris G. R. (Thousand Oaks, CA: Sage), 183–192.

[B64] Slaughter J. E. Zickar M. J. Highhouse S. Mohr D. C. (2004). Personality trait inferences about organizations: development of a measure and assessment of construct validity. J. Appl. Psychol. 89, 85–103. 10.1037/0021-9010.89.1.85 14769122

[B65] Spence M. (1973). Job market signaling. Q. J. Econ. 87, 355–374. 10.2307/1882010

[B66] Stevens C. K. Kristof A. L. (1995). Making the right impression: a field study of applicant impression management during job interviews. J. Appl. Psychol. 80, 587–606. 10.1037/0021-9010.80.5.587

[B67] Stevens C. K. Mitchell T. R. Tripp T. M. (1990). Order of presentation and verbal recruitment strategy effectiveness. J. Appl. Soc. Psychol. 20, 1076–1092. 10.1111/j.1559-1816.1990.tb00391.x

[B68] Taylor M. S. Bergmann T. J. (1987). Organizational recruitment activities and applicants' reactions at different stages of the recruitment process. Pers. Psychol. 40, 261–285. 10.1111/j.1744-6570.1987.tb00604.x

[B69] Thompson E. R. (2007). Development and validation of an internationally reliable short-form of the positive and negative affect schedule (PANAS). J. Cross Cult. Psychol. 38, 227–242. 10.1177/0022022106297301

[B70] Tofighi D. MacKinnon D. P. (2011). RMediation: an R package for mediation analysis confidence intervals. Behav. Res. Methods 43, 692–700. 10.3758/s13428-011-0076-x 21487904 PMC3233842

[B71] Truxillo D. M. Bauer T. N. (2011). Applicant reactions to organizations and selection systems, in APA Handbook of Industrial and Organizational Psychology, ed Zedeck S. (Washington, DC: American Psychological Association), 379–397.

[B72] Truxillo D. M. Bauer T. N. McCarthy J. M. Anderson N. R. Ahmed S. M. (in press). Applicant perspectives on employee selection systems, in Handbook of Industrial, Work, Organizational Psychology, eds Ones D. S. Anderson N. Sinangil H. K. Viswesvaran C. (Thousand Oaks, CA: Sage).

[B73] Truxillo D. M. Bodner T. E. Bertolino M. Bauer T. N. Yonce C. A. (2009). Effects of explanations on applicant reactions: a meta-analytic review. Int. J. Select. Assess. 17, 346–361. 10.1111/j.1468-2389.2009.00478.x

[B74] Tsai W.-C. Huang T.-C. (2014). Impression management during the recruitment process, in The Oxford Handbook of Recruitment, eds Yu K. Y. T. Cable D. M. (New York, NY: Oxford University Press), 314–334.

[B75] Turban D. B. Dougherty T. W. (1992). Influences of campus recruiting on applicant attraction to firms. Acad. Manage. J. 35, 739–765. 10.2307/256314

[B76] Wanous J. P. (1976). Organizational entry: from naive expectations to realistic beliefs. J. Appl. Psychol. 61, 22–29. 10.1037/0021-9010.61.1.22

[B77] Wanous J. P. Poland T. D. Premack S. L. Davis K. S. (1992). The effects of met expectations on newcomer attitudes and behaviors: a review and meta-analysis. J. Appl. Psychol. 77, 288–297. 10.1037/0021-9010.77.3.288 1534799

[B78] Watson D. Clark L. A. Tellegen A. (1988). Development and validation of brief measures of positive and negative affect: the PANAS scales. J. Pers. Soc. Psychol. 54, 1063–1070. 10.1037/0022-3514.54.6.1063 3397865

[B79] Wilhelmy A. Kleinmann M. König C. J. Melchers K. G. Truxillo D. M. (2016). How and why do interviewers try to make impressions on applicants? A qualitative study. J. Appl. Psychol. 101, 313–332. 10.1037/apl0000046 26436440

[B80] Williams L. J. O'Boyle E. H. Jr. (2008). Measurement models for linking latent variables and indicators: a review of human resource management research using parcels. Hum. Resour. Manage. Rev. 18, 233–242. 10.1016/j.hrmr.2008.07.002

