# Supplementary material for: Selling and Smooth-Talking: Effects of Interviewer Impression Management from a Signaling Perspective
Source: Front Psychol. 2017 May 29;8:740. doi: 10.3389/fpsyg.2017.00740 (PMC5447071; doi:10.3389/fpsyg.2017.00740)
Supplement: Supplementary file 1 [file DataSheet1.docx]

Appendix A

**Sample questions from the interview guide sorted by topical areas**

1. Interest in psychology

*How would you explain what psychology actually is to someone who is not familiar with psychology?*

1. Realistic expectations regarding content and later occupation

*How do you envision your future professional occupation?*

1. Commitment

*Was there a period in your life in which you were especially burdened (in the sense of having a lot to do or having to deal with many things at the same time)? How did you deal with this?*

1. Professional attitude

*How do you define yourself (your role) as a psychologist in problem solving?*

1. Social skills

*How would others (e.g., good friends, peers, colleagues) describe you? Is there a difference between your own description and that of others? If so, how do you explain this?*

1. Interest in interdisciplinary collaboration

*Can you think of specific fields of work where an interdisciplinary team would be ideal?*

Appendix B

**Survey Items**

| Perceived organization-enhancement (T2) |
| --- |
| 1. The interviewers attempted to present the study program in a positive way. |
| 2. The interviewers expressed their enthusiasm about [name of university]. |
| Perceived applicant-enhancement (T2) |
| 1. The interviewers complimented me. |
| 2. The interviewers indicated that I was a prime candidate for this study program. |
| 3. The interviewers praised me. |
| Organizational prestige (T1 and T2) |
| 1. Students are probably proud to say they study at this university. |
| 2. This is a reputable university to study at. |
| 3. This university probably has a reputation as having excellent study programs. |
| 4. There are probably many who would like to study at this university. |
| Acceptance intention (T1 and T2) |
| 1. How likely are you to accept an offer from this university based on the information you have so far? |
| Positive affect (T1 and T2) |
| At the moment, I’m feeling… |
| 1. Active |
| 2. Inspired |
| 3. Alert |
| 4. Determined |
| 5. Attentive |
| Interview self-efficacy (T1 and T2) |
| 1. I believe I can perform well in interviews. |
| 2. I am not good at performing well at interviews like this. |
| 3. I am confident in my abilities regarding interviews. |
| Interview experience (T1) |
| 1. How many prior interviews have you had in your life? |
